# Differences in characteristics and interactions with close contacts among PWID in the San Diego Border Region before and during the COVID-19 pandemic

**DOI:** 10.1016/j.jmh.2024.100267

**Published:** 2024-09-24

**Authors:** Lara K Marquez, Natasha K Martin, Steffanie A Strathdee, Britt Skaathun

**Affiliations:** Division of Infectious Diseases and Global Public Health, University of California San Diego, 9500 Gilman Drive, La Jolla 92037, CA, USA

**Keywords:** Substance use, Social networks, COVID-19 pandemic

## Abstract

**Background:**

Travel restrictions implemented to mitigate the spread of SARS-CoV-2 decreased mobility and reduced physical contact during 2020–2021 for many in the general population. This analysis explored changes to network contacts among people who inject drugs (PWID) in the San Diego Border Region (SDBR) by cross-border mobility before and during the COVID-19 era.

**Methods:**

Baseline data collected between October 2020–2021, from a cohort study of PWID in the SDBR were used to retrospectively describe differences in baseline characteristics across cross-border PWID groups (cross-border PWID [CB-PWID]: *n* = 206; San Diego PWID [SD-PWID]: *n* = 203; Tijuana PWID [TJ-PWID]: *n* = 202). Chi-square and Fisher's exact tests evaluated sociodemographic, injecting risk behaviors, harm reduction service history, incarceration history, non-fatal overdose, HCV, HIV. Median differences in sex, drug/alcohol, and close partners before and during the pandemic among all PWID and by cross-border PWID status were evaluated using Kruskal-Wallis tests. Pairwise associations across cross-border PWID groups were assessed using the Dwass, Steel, Critchlow-Fligner multiple comparison test.

**Results:**

Among 611 PWID, the number of sex, drug/alcohol-related partners and close contacts before and during the pandemic remained relatively stable (p_sex_=0.71;p_drug/alcohol_=0.15;p_close_=0.09). PWID in San Diego experienced the greatest difference in drug/alcohol-related partners (median[IQR]:-1[-6,0]), while cross-border PWID reported the smallest change in close contacts versus pre-pandemic (median[IQR]:0[0,1]). PWID in Tijuana had the greatest proportion (87%) of close contacts who injected drugs of all three groups.

**Conclusions:**

Compared to pre-pandemic, the median number of sex partners, drug/alcohol-related partners, and close contacts remained stable among PWID in the SDBR. Future research should explore how these network contacts evolve over time.

## 1 Introduction

The COVID-19 pandemic epitomizes how sudden disruptions can impact vulnerable populations such as people who inject drugs (PWID), by increasing housing instability (Croxford et al., 2021), risk of overdose (Stephenson, 2021; Government of Canada, 2021) poorer mental health outcomes, reduced provision and utilization of harm reduction services (Aponte-Melendez et al., 2021) and overall mortality (Wang et al., 2021). Injecting and sexual network data can provide insight on social and interpersonal factors which impact both drug use practices and infectious disease transmission among PWID (Friedman et al., 1997; Suh et al., 1997; Weeks et al., 2002; Valente and Vlahov, 2001; Kirst, 2009). As the COVID-19 era has resulted in abrupt and widespread disruption of mobility and social interactions (Moreland et al., 2020), the impact on injecting and sexual networks among PWID continues to be studied. Recent evidence has increased concern for unsafe injecting practices due to interruptions in harm reduction services (Aponte-Melendez et al., 2021) and reduction in social interactions, as has been shown historically (Simmons and Singer, 2006). Though the impact on injecting partners is less clear, decreases in the number of sexual partners (Grov et al., 2022) in the United States (US) have also been reported during the COVID-19 pandemic.

Spanning San Diego, US, and Tijuana, Mexico, the San Diego Border Region (SDBR), is located along a major drug trafficking route (Bucardo et al., 2005) where PWID frequently crossed to inject drugs from San Diego to Tijuana in the pre-COVID-19 pandemic era, as drugs are thought to be more available and less expensive (Borquez et al., 2019; Volkmann et al., 2012; Wagner et al., 2012). Travel restrictions implemented to mitigate the spread of SARS-CoV-2 have led to decreased mobility and consequently a reduction in the number of physical contacts within the last year for many in the general population (Moreland et al., 2020). On March 23, 2020, the US-Mexico border closed to non-essential travel and has remained closed through November 8, 2021 (U.S. Customs and Border Protection, 2020) though it was unclear if or how these changes during the pandemic impacted PWID on both sides of the US-Mexico border.

In October 2020, the La Frontera Study began recruiting participants from the SDBR for a longitudinal cohort study analyzing trends in regional populations of PWID who cross the US-Mexico border to use illicit drugs (i.e., cross-border PWID) and its impact on HIV, hepatitis C virus (HCV), and SARS-CoV-2 transmission and overdose (Strathdee et al., 2021). In the early COVID-19 pandemic era, cross-border travel was restricted, and overall mobility bi-directionally decreased (U.S. Customs and Border Protection, 2020) but the impact of these restrictions on cross-border and in-country PWID network contacts was unknown. This analysis sought to describe baseline characteristics of PWID in the SDBR by cross-border mobility and explored the composition and changes to PWID network contacts during the COVID-19 pandemic era.

## 2 Material and methods

### 2.1 Study population and procedures

Baseline data for this descriptive analysis were collected as part of a longitudinal study, among PWID in the SDBR between October 2020 and October 2021, as previously described (Strathdee et al., 2021). Three groups of PWID aged 18 years or older, were recruited using targeted sampling in San Diego, USA and Tijuana, Mexico: (1) PWID who reported crossing the border from San Diego to inject drugs in Tijuana within the last 2 years (CB-PWID); (2) PWID who are living in San Diego and do not report crossing the border to inject drugs in Tijuana (SD-PWID), and (3) PWID who are living in Tijuana (TJ-PWID). Cross-border PWID were recruited and interviewed in person in Tijuana at baseline to confirm that they cross the border from San Diego. PWID were considered non-cross-border PWID if they lived in either San Diego or in Tijuana and reported never using illicit drugs across the US-Mexico border. An in-person supplemental network survey containing detailed questions on partners was offered to all participants at a supplemental visit two weeks post baseline. This study was approved by the University of California San Diego Institutional Review Board and Xochicalco University.

### 2.2 Measures

#### 2.2.1 Sociodemographic characteristics

Sociodemographic characteristics including age, gender, ethnicity, and age at first injection were assessed at baseline. For this descriptive analysis, gender was re-categorized as cisgender man, cisgender woman, and transgender/nonbinary.

#### 2.2.2 Injection risk behavior

HIV and HCV-associated injection risk behaviors were assessed in the last 6 months during the pandemic and as a multilevel variable (more likely/less likely/same than prior to the pandemic) to evaluate the likelihood of injecting alone compared to pre-pandemic. Distributive syringe sharing and receptive syringe sharing were dichotomized from a categorical frequency variable (at least once in the last 6 months/never in the last 6 months).

#### 2.2.3 Harm reduction services

Utilization and history of harm reduction services including the number of sterile needles/syringes obtained from a needle/syringe program (NSP) in the last 6 months during the pandemic, lifetime history of enrollment in a substance use clinic, a buprenorphine/suboxone drug treatment program (ever/never) and current enrollment into a methadone program (yes/no) were also evaluated.

#### 2.2.4 Incarceration

Lifetime and recent (last 6 months) history of incarceration in a prison, jail was assessed by a dichotomous variable (yes/no). History of being held in a detention center was dichotomized for lifetime history (yes/no) and reported as a frequency for history of detention in the last 6 months. Prisons were distinguished from jails in that prison referred to a federal level institution and jail referred to a state- or County-level institutions, regardless of whether PWID were held with or without sentencing. Detention centers were defined as a place where a person could be held for a maximum of 72 hours or a center where migrants are held in Mexico during the processing and resolution of political asylum cases in the US.

#### 2.2.5 Non-fatal overdose

Overdose was assessed as a dichotomous variable for lifetime history (ever/never) and as a frequency of occurrence in the last 6 months.

#### 2.2.6 HCV and HIV

HCV and HIV seropositivity were dichotomized to indicate whether participants received a positive result after two rapid serotests—first, with Miriad® HIV/HCV Antibody InTec Rapid Anti-HCV Test (Avantor, Radnor, PA) and second, with either Oraquick® HIV or Oraquick® HCV (Orasure, Bethlehem, PA).

#### 2.2.7 Number of contacts pre-pandemic

To capture the number of people that PWID would normally have had close contact in the pre-pandemic era, participants were asked to remember the first two weeks of February 2020, a typical 14-day period before the pandemic, and report the number of people that they would have normally had close contact with during that time. Close contact was defined as either physical contact with an individual or non-physical (face-to-face) contact within 6 feet that lasted more than one hour. Participants were able to use their phone and/or calendar to remind them of who they may have been in contact with. Participants were also asked to report the number of these contacts who they may have had sex with during that time and how many they used drugs with.

#### 2.2.8 Number of contacts during the pandemic

To capture the number of contacts during the pandemic, PWID were asked to report the number of sex/romantic partners and drug/alcohol-related partners that they had close contact with in the past 14 days. Close contact was defined the same as for pre-pandemic. The total number of close contacts was the sum of all partner types reported, which were not limited to sex/romantic partners or drug/alcohol-related partners (i.e., close friends, family-relatives by blood or marriage, roommates, neighbors, work related, classmates, people who provide you a service, and others). Participants were also asked to estimate the number of close contacts who they have lost touch with because of the pandemic and to list the number of friends or people they associate with and talk to about things that are important to them that they have seen in the last 30 days. A maximum of 20 names were allowed to be listed. Participants were requested to list each name in order of closeness (starting with the first closest partner – defined as a partner closest to them who they talked to about things that are important to them and have seen in the last 30 days). Detailed information on partner type, sexual relationship, and questions regarding injection drug use, distributive and receptive injection equipment sharing were asked for the first five close partners listed.

### 2.3 Statistical analysis

Univariate descriptive statistics were used to evaluate sample characteristics among all PWID and by CB-PWID status (CB-PWID, SD-PWID, TJ-PWID). Categorical variables were assessed using Chi-square and Fisher's exact tests and non-normally distributed, continuous variables were assessed using the Kruskal-Wallis test for nonparametric data to determine associations between CB-PWID status and sociodemographic characteristics, injecting risk behaviors, harm reduction service history, incarceration history, non-fatal overdose, HCV, and HIV. Pairwise comparisons were evaluated for significance using the Dwass, Steel, Critchlow-Fligner statistic.

Median numbers of partners before and during the pandemic were compared across groups and by gender (cisgender man/woman, transgender/nonbinary) and changes in the median number of drug/alcohol-related partners, sex partners, and close contacts pre-pandemic versus during pandemic, expressed as median differences, were evaluated using the Kruskal-Wallis test. Diversity in closest partner type and by partner type who use drugs by injection was assessed across CB-PWID groups using Simpson's Diversity Index (Simpson, 1949). Diversity indices were tested for statistical significance using the Chi-square test. Associations were considered statistically significant if *p* < 0.05. All statistical analysis were performed in SAS version 9.4 (Cary, NC).

## 3 Results

### 3.1 Demographic characteristics

Demographic characteristics of PWID in the SDBR are shown by CB-PWID status in Table 1. Among 611 PWID in the SDBR, 206 were CB-PWID, 203 were SD-PWID, and 202 were TJ-PWID. The median age of PWID was 43 years old [Interquartile Range, IQR: 35–52] and 72% identified as Hispanic, Latinx or Mexican. Non-cross border PWID were the oldest (median [IQR]: 44 [38–53]) across CB-PWID groups. Though the median age at first injection did not significantly differ across groups, age at first injection was similar across all groups (median [IQR] among all PWID: 20 [17–26]).

**Table 1:** Demographic characteristics of PWID in the San Diego Border Region by cross-border PWID status.

Characteristic	Cross-Border PWID (*n* = 206)	San Diego PWID (*n* = 203)	Tijuana PWID (*n* = 202)	Total *N* = 611	P-value**	P_1,2_	P_1,3_	P_2,3_
Age, *Median (IQR)*	41 (35, 51)	40 (31, 53)	44 (38, 53)	43 (35, 52)	0.03	0.95	0.05	0.09
Cisgender woman, *n (%)*	44 (21.4)	57 (28.1)	55 (27.2)	156 (25.5)	0.05	–	–	–
Hispanic/Latinx/Mexican, *n (%)*	156 (75.7)	89 (43.8)	195 (96.5)	440 (72.0)	<0.0001	–	–	–
Age at first injection, *Median (IQR)*	20 (17, 25)	20 (17, 27)	20 (17, 26)	20 (17, 26)	0.51	0.54	0.63	0.97
**Injection risk behavior, past 6 months, *n (%)***								
Likelihood of injecting alone in past 6 months compared to pre-pandemic								
More likely	26 (12.7)	32 (16.0)	14 (7.0)	72 (11.9)	<0.0001	–	–	–
Less likely	76 (37.1)	51 (25.5)	43 (21.4)	170 (28.1)				
Same	103 (50.2)	117 (58.5)	144 (71.6)	364 (60.1)				
Distributive syringe sharing at least once	102 (49.5)	85 (41.9)	157 (77.7)	344 (56.3)	<0.0001	–	–	–
Receptive syringe sharing at least once	95 (46.1)	83 (40.9)	139 (68.8)	317 (51.9)	<0.0001	–	–	–
								
**Harm reduction service history**								
Number of sterile needles/syringes obtained from NSP in last 6 months, *Median (IQR)*	20 (2.5, 40)	25 (4, 40)	3 (2, 8.5)	8.5 (3, 30)	0.0004	0.98	0.03	0.001
*Range*	0–200	0–120	0–30	0–200				
Ever attended a substance use clinic, *n (%)*	125 (60.7)	59 (29.1)	46 (22.8)	230 (37.6)	0.004	–	–	–
Ever enrolled in buprenorphine/ suboxone program, *n (%)*	10 (4.9)	29 (14.3)	1 (0.5)	40 (6.5)	<0.0001	–	–	–
Ever enrolled in methadone treatment program, *n (%)*	34 (16.5)	58 (28.6)	48 (23.8)	140 (22.9)	0.36	–	–	–
Currently enrolled in methadone treatment program, *n (%)*	11 (5.3)	20 (9.9)	4 (2.0)	35 (5.7)	<0.0001	–	–	–
**Overdose**								
Ever overdosed, *n (%)*	102 (50.0)	112 (55.2)	103 (51.0)	317 (52.0)	0.52	–	–	–
Number of times overdosed in life, *Median (IQR)*	0 (0, 3)	1 (0, 3)	1 (0, 2)	1 (0, 3)	0.21	0.38	0.95	0.21
Number of times overdosed in last 6 months, *Median (IQR)*	0 (0, 1)	0 (0, 1)	0 (0, 1)	0 (0, 1)	0.20	0.30	0.99	0.25
**Incarceration history, *n (%)***								
History of being in prison	90 (43.7)	80 (39.6)	123 (60.9)	293 (48.0)	<0.0001	–	–	–
History of being in jail	84 (40.8)	127 (62.9)	83 (41.1)	294 (48.2)	<0.0001	–	–	–
Been in prison/jail in last 6 months	39 (18.9)	15 (7.4)	7 (3.5)	61 (10.0)	0.33	–	–	–
History of being in detention center	72 (21.1)	27 (18.5)	49 (39.8)	148 (24.3)	<0.0001	–	–	–
Number of times in detention center in last 6 months, *Median (IQR)*	2 (1, 3)	0 (0, 1)	2 (0, 4)	1 (0, 3)	0.0004	0.001	0.94	0.001
**HCV and HIV characteristics, *n (%)***								
Ever received HCV test	111 (53.9)	164 (81.6)	81 (40.3)	356 (58.6)	<0.0001	–	–	–
Received last HCV test after border closure (March 23,2020)	54 (26.2)	65 (32.0)	30 (14.9)	149 (24.4)	0.0002	–	–	–
HCV seropositivity	68 (33.0)	102 (50.3)	71 (35.2)	241 (39.4)	0.001	–	–	–
HIV seropositivity	8 (3.9)	7 (3.5)	32 (15.8)	47 (7.7)	<0.0001	–	–	–
HCV/HIV seropositivity	4 (1.9)	2 (1.0)	11 (5.5)	17 (2.8)	0.02	–	–	–

Cross-Border PWID refers to PWID who cross the US-Mexico border to inject drugs; Non-Cross-Border PWID refers to people who inject drugs in their city of residence (either San Diego or Tijuana) and do not cross the border to inject drugs. IQR: interquartile range. *Of those previously diagnosed with HCV; **Overall p-value; P_1,2_: P-value for Dwass, Steel, Critchlow-Fligner multiple comparison procedure between CB-PWID and SD-PWID; P_1,3_: P-value for CB-PWID versus TJ-PWID; P_2,3_: P-value for SD-PWID versus TJ-PWID.

### 3.2 Injecting risk behaviors during the pandemic

**Fig. 1:**
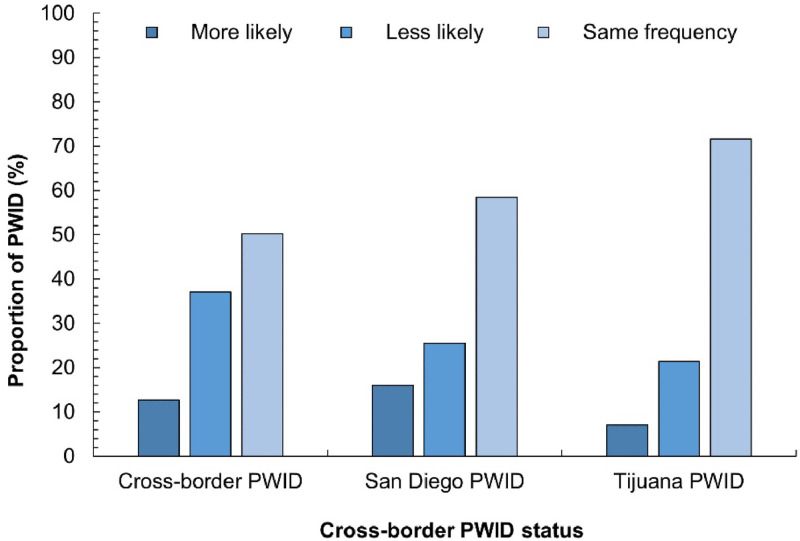
Likelihood of PWID injecting alone in the past 6 months during the pandemic compared to pre-pandemic by cross-border PWID status. PWID: people who inject drugs.

**Fig. 2:**
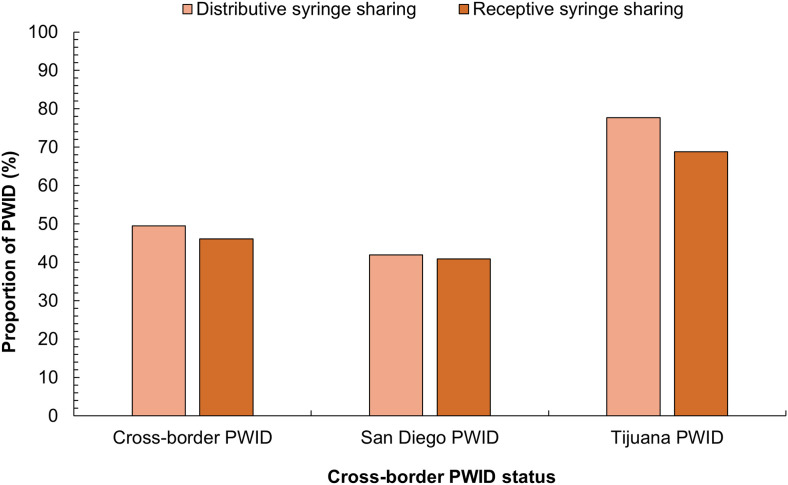
Distributive and receptive syringe sharing behaviors in the last 6 months by cross-border PWID status.

The likelihood of injecting alone in the past 6 months during the pandemic compared to pre-pandemic significantly differed (Fig. 1; *p* < 0.0001). Most PWID continued to inject alone at the same frequency (60.1%) or were less likely to inject alone (28.1%) in the past 6 months compared to pre-pandemic. TJ-PWID reported the fewest changes in this behavior as 71.6% continued to inject alone at the same frequency compared to pre-pandemic and to 50.2% of CB-PWID and 58.5% of SD-PWID. Furthermore, 37.1% of CB-PWID were less likely to inject alone compared to pre-pandemic and to 25.5% of SD-PWID and 21.4% of TJ-PWID. Overall, 56.3% of PWID reported engaging in distributive syringe sharing and 51.9% in receptive syringe sharing at least once in the last 6 months (*p* < 0.0001; Fig. 2). SD-PWID reported the lowest rates of distributive and receptive syringe sharing in the last 6 months of all groups (41.9% and 40.9%, respectively), whereas TJ-PWID reported the highest rates (77.7% and 68.8%, respectively).

### 3.3 Harm reduction services

Across all harm reduction services including utilization of NSPs and history of enrollment in drug treatment programs, TJ-PWID reported the lowest utilization rates, except for history of enrollment in a methadone treatment program (Table 1). The median number of sterile syringes/needles obtained from a NSP in the last 6 months during the pandemic significantly differed across groups (*p* = 0.0004), with TJ-PWID reporting by far the lowest sterile syringes/needles received (median [IQR]: 3, 2–8.5) compared to 20 [IQR: 2.5–40] sterile syringes/needles from CB-PWID and 25 [IQR: 4–40] among SD-PWID. CB-PWID had the greatest proportion of PWID with a history of attending a substance use clinic (60.7%), whereas TJ-PWID had the lowest history of attendance (22.8%; *p* = 0.004). Furthermore, TJ-PWID had the lowest proportion of PWID with a history of enrollment in buprenorphine/suboxone programs whereas SD-PWID had the highest (14.3% vs 0.5%). Few PWID (5.7%) were currently enrolled in a methadone treatment program.

### 3.4 Non-fatal overdose

Overall, history of non-fatal overdose was similar across CB-PWID groups with 52% of all PWID in the SDBR reporting a history of non-fatal overdose (Table 1). SD-PWID reported the greatest frequency of lifetime non-fatal overdoses (median [IQR]: 1 [0–3]). Similarly, frequencies of non-fatal overdoses were observed across all groups in the last 6 months, as PWID reported no non-fatal overdoses (median [IQR]: 0 [0–1]).

### 3.5 Incarceration history

History of incarceration significantly differed across CB-PWID groups (Table 1). TJ-PWID had the greatest proportion with a history of imprisonment (60.9%), whereas SD-PWID reported the greatest proportion with a history of being in jail (62.9%). TJ-PWID also reported both the greatest proportion of PWID with a history of being in a detention center (39.8%) and had been detained the most in the last 6 months (median times detained in last 6 months [IQR]: 2 times [0–4]) compared to 18.5% of SD-PWID (median [IQR]: 0 [0–1]; *p* = 0.001) and to 21.1% of CB-PWID (median [IQR]: 2 [1–3]; *p* = 0.001). Recent history of being in jail or prison in the last 6 months did not significantly differ across CB-PWID groups (*p* = 0.33).

### 3.6 HCV and HIV testing and seroprevalence

Of 608 PWID, 58.6% had ever received an HCV test, with 24.4% reporting their last HCV test since the border closure on March 23, 2020 (Table 1). Among all PWID (*N* = 611), HCV seroprevalence was 39.4%, HIV seroprevalence was 7.7%, and HCV/HIV coinfection seroprevalence was 2.8%. HCV seropositivity was highest among SD-PWID (50.3%). TJ-PWID reported the lowest rates of HCV testing (40.3%) of all the groups, including during the pandemic (14.9%).

### 3.7 Pandemic differences in number of partners

**Fig. 3:**
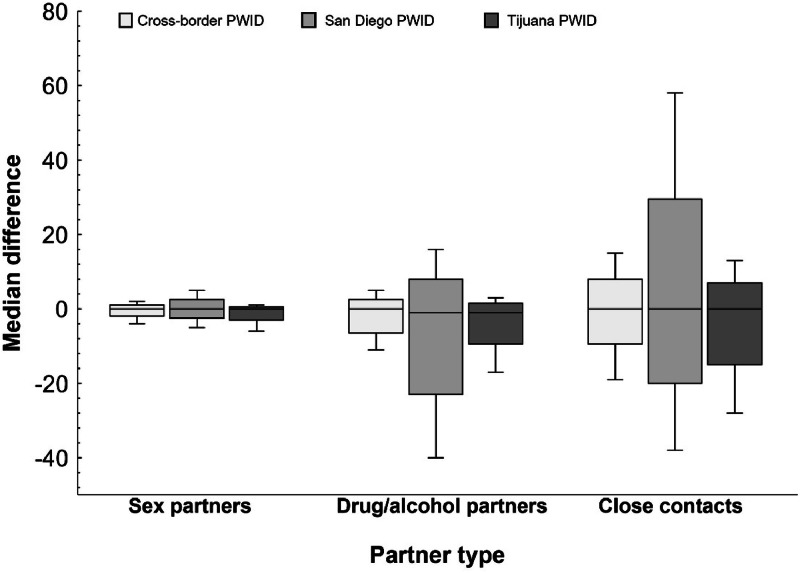
Median difference in the number of partners reported in two-week period pre-pandemic vs during pandemic among PWID by cross-border PWID status. PWID: people who inject drugs.

Median differences in the number of sex, drug/alcohol and close partners reported in the two-week period pre-pandemic (February 2020) and during the pandemic by CB-PWID status are shown in Fig. 3. While median differences in the number of sex partners, drug/alcohol-related partners and close contacts did not significantly differ across CB-PWID groups (psex: 0.71; pdrug/alcohol: 0.15, pclose: 0.09, respectively), SD-PWID reported the greatest differences in drug/alcohol-related partners (median [IQR]: −1 [−6,0]) compared to the CB-PWID (median [IQR]: 0 [−2,0]) and to TJ-PWID (median [IQR]: −1 [−2,0]). Further, non-cross border PWID in San Diego (SD-PWID) and in Tijuana (TJ-PWID) reported similar median differences in the number of close contacts (median [IQR]: −2 [0,1]) compared to CB-PWID (median [IQR]: 0 [0–1]). SD-PWID also experienced greater overall variation in the number of close network contacts than CB-PWID and TJ-PWID. Median differences were not significant (*p* > 0.20) when stratified by gender (data not shown).

### 3.8 Injecting and sexual contacts during the pandemic

**Fig. 4:**
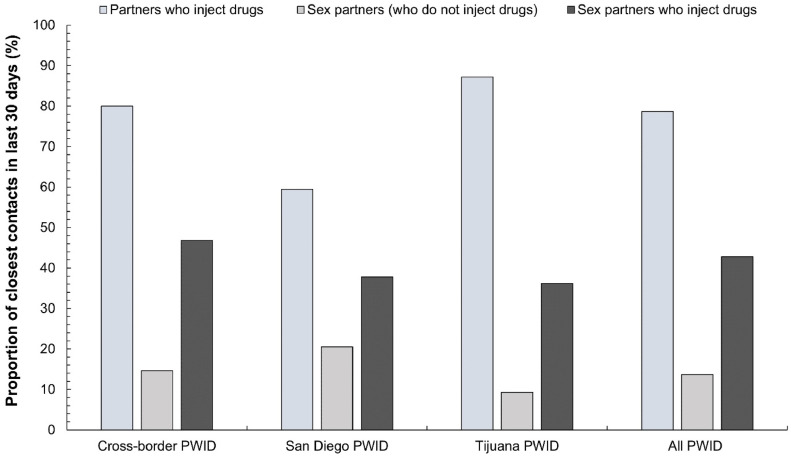
Proportion of closest contacts who PWID reported injecting drugs with and engaging in sex with during the pandemic by cross-border PWID group. PWID: people who inject drugs.

The proportion of top closest contacts during the pandemic by injecting and sexual network contact status is shown across CB-PWID groups in Fig. 4. Most close partners (78.7%) reported by PWID in the SDBR were partners who used drugs by injection. Few partners were exclusively sex partners who did not use drugs by injection (13.7%). Overall, TJ-PWID had the greatest proportion of close partners who used drugs by injection (87.2% vs. 80.0% among CB-PWID and 59.4% among SD-PWID; *p* < 0.001). While SD-PWID had the greatest proportion of sex partners who did not use drugs by injection (20.5% vs 14.6% among CB-PWID and 9.3% among TJ-PWID; *p* = 0.01), CB-PWID had the greatest proportion of close sex partners who used drugs by injection (47% compared to 38% among SD-PWID and 36% among TJ-PWID; *p* = 0.33).

### 3.9 Partner types who use drugs by injection

Among all PWID, 79% of the top five closest partners used drugs by injection (**Supplementary Table 2**). Among all partners who used drugs by injection, 15% were also sex partners, 35% were described as drug/alcohol-related partners, and 50% were friends. TJ-PWID and CB-PWID reported the greatest proportion of close contacts who use drugs by injection (87% and 80%, respectively). Across all groups, the greatest proportion of close partner type who used drugs by injection were described as friends. Among CB-PWID, 41.7% of closest contacts who use drugs by injection were described as friends, while TJ-PWID more evenly described these close contacts as drug/alcohol partners (37.0%) or as friends (39.7%). SD-PWID reported the highest proportion of close partners who used drugs by injection and who were also sex partners (16.0%). The proportion of closest partners who use drugs by injection are shown by CB-PWID status in **Supplementary Table 3**. A greater number of close contacts who both used drugs by injection and who encouraged the respondent to inject drugs were also considered friends rather than drug/alcohol-related partners, with the key difference between these classifications being that PWID knew the friend longer or felt closer to them.

### 3.10 Diversity of partner types during the pandemic

Diversity of close partner types during the pandemic are shown in Table 2. There was moderate diversity in close partner types reported during the pandemic among CB-PWID (Simpson's D: 0.65) and slightly higher diversity among non-cross border PWID (SD-PWID: 0.72; TJ-PWID: 0.70; *p* = 0.10). SD-PWID also had the highest diversity in partner types who use drugs by injection (Simpson's D: 0.66), whereas CB-PWID and TJ-PWID had similar diversity indices (Simpson's D: 0.59 and 0.60, respectively; *p* = 0.48).

**Table 2:** Diversity indices for close partner types during the pandemic among PWID in the San Diego Border Region (*N* = 611).

Diversity measures	Cross-border PWID (*n* = 206)	San Diego PWID (*n* = 203)	Tijuana PWID (*n* = 202)	P-value
All partner types	0.65	0.72	0.70	0.10
Partner types who inject drugs	0.58	0.66	0.60	0.48

Diversity indices were calculated using Simpson's Diversity Index (range: 0-1). PWID: people who inject drugs. Partner types included romantic/sex partner, friend, neighbor/housemate, coworker/boss, family-relative (by blood or marriage), drug/alcohol-related, other.

## 4 Discussion

This analysis provides preliminary insights into differences between CB-PWID groups and changes in close partner types among PWID in the SDBR. Our analysis showed that TJ-PWID had the highest proportion of PWID with stable frequency of injecting alone compared to pre-pandemic, and who engaged in distributive and receptive syringe sharing in the last 6 months during the pandemic. Conversely, SD-PWID had the greatest proportion of people in their network who do not inject drugs (40.6%), reported the lowest proportion of distributive and receptive syringe sharing (41.9% and 40.9%, respectively) and the highest rates of historical HCV testing (81.6%). Further, TJ-PWID reported the lowest recent utilization of NSPs and HCV testing and the highest HIV seropositivity (15.8%) and HCV/HIV coinfection seropositivity (5.5%) whereas SD-PWID had the greatest HCV seropositivity (50.3%). Compared to both historical and recent studies which reported HCV seroprevalence >90% in Tijuana (White et al., 2007; Fleiz et al., 2019), current estimates of HCV seroprevalence among PWID are lower (at 35%). However, we believe that this may be a result of a difference in study populations of PWID, as previous studies had fewer cisgender women in Tijuana participating (8% in (White et al., 2007) and 10% in (Fleiz et al., 2019) compared to 26% in our analysis) and women have a lower HCV seroprevalence in Tijuana. Additionally, our HCV seropositivity among San Diego CB-PWID was more consistent with a previous HCV seroprevalence estimate among PWID in San Diego between 2012 and 2014 (50% vs. 66% in (Horyniak et al., 2017)).

Our analysis of network contacts showed that SD-PWID experienced a greater reduction in drug/alcohol-related partners and close contacts pre-pandemic compared to during the pandemic which suggests that their networks may have been more greatly impacted by restrictions placed in San Diego and in California during the pandemic compared to other PWID who either continued to cross the border during the pandemic or who lived in Mexico. Within a network, having a greater number of injecting partners has been associated with increased injecting frequency and injecting risk behaviors and subsequently associated with increased risk of HCV (Spelman et al., 2019). Given that SD-PWID had the lowest proportion of people in their network who inject drugs, but the greatest HCV seropositivity across all groups, the impact of norms in the injecting network on injecting risk behaviors should be further explored.

Additionally, many close contacts who use drugs by injection and engage in receptive or distributive syringe sharing were described as friends and not as a drug/alcohol-related partners, regardless of CB-PWID status. As diversity was moderately high across all CB-PWID groups, most close partners who used drugs by injection and who encouraged the respondent to inject drugs were considered friends. Thus, peer-led efforts and organizations offering harm reduction, HIV, and HCV prevention services may be most successful in preventing future infections (Weeks et al., 2009; Lazarus et al., 2016) and overdose (Marshall et al., 2017), as this approach offers numerous benefits to the PWID community such as mediating stigma (Damon et al., 2017), increasing self-empowerment (Damon et al., 2017; Lazarus et al., 2014), offering employment opportunities (Lazarus et al., 2014), and reducing initiation of drug use (Richardson et al., 2014).

### 4.1 Comparisons with existing literature

While implications from the pandemic are still being studied, our results showing stable network contacts before and during the pandemic among PWID in the SDBR support a previous analysis by Strathdee et al. which reported lower social distancing (46.5%) among current PWID (Strathdee et al., 2021). However, our analysis showed no significant difference in sex contacts before and during the pandemic, whereas in an online, cross-sectional study from the US showed a reduction in sex partners during the early pandemic (April-May 2020) among cisgender men, transgender men and women and men who have sex with men (Grov et al., 2022). As our analysis spanned a greater time period and recorded recent information on sexual network contacts to compare to the number of sexual network contacts pre-pandemic, it is possible that we do not capture the immediate impact in sexual network contacts in the first few months of the pandemic. However, our approach captures variations in changes to these network contacts throughout the course of the pandemic thus far. Additionally, we observed similar high frequencies of distributive and receptive syringe sharing (56.3% and 51.9%, respectively) in the overall PWID cohort, though slightly greater than the 46% of PWID who reported direct sharing of needles, syringes, and other injecting paraphernalia in a cross-sectional survey of PWID in England, Wales, and Northern Ireland between June and October of 2020 (Croxford et al., 2021).

### 4.2 Limitations

We acknowledge that our study has the following limitations. First, more detailed data was collected on the first five closest partners listed by each participant within the last 30 days during the pandemic. Thus, this does not capture all possible partners of the participant as they may engage in other high-risk behaviors such as injecting drugs or having sex with other partners outside of this group. As we do not ask participants for an exhaustive list of partners, we are also unable to report the overall network size. However, we believe that those listed as closest contacts are those who they have interacted with most frequently and thus could represent the greatest, current cumulative risk profile for the participant. Future analysis should explore overall network size across CB-PWID groups.

Second, our survey includes questions that were asked over a 6-month time frame and/or the last 30 or 14-day period to capture the number and partner type of network contacts. We recognize that this can only provide a snapshot of network contacts at that unique point during the pandemic and is not an exhaustive data capture of their network contacts over the entire pandemic period, however as follow-up data collection occurs every 6 months, we will be able to track changes throughout the pandemic period.

As subsequent data collection visits are completed, diversity of partners, the proportion of partners who use drugs by injection, HCV and HIV characteristics can be further evaluated to determine whether diversity changes over time.

### 4.3 Conclusions

This analysis provides preliminary evidence that the number of network contacts remained relatively stable among PWID in the SDBR. Further, the high frequency of friends who use drugs by injection and engage in distributive and receptive syringe sharing highlight the importance of peer-led disease and harm reduction prevention and intervention services. Future research should explore how these network contacts and diversity of network composition evolve over time.

## Ethics approval and consent to participate

Protocols were approved by the Human Research Protections Program and Biosafety Committee at the University of California San Diego (UCSD) and the institutional review board at Xochicalco University.

## Availability of data and materials

The datasets used and/or analyzed during the current study are available from the Principal Investigator of the La Frontera Study (SAS) on reasonable request.

## Funding

This work was supported by the 10.13039/100000002National Institutes of Health (NIH)-National Institutes of Drug Abuse (NIDA) R01DA049644. Marquez LK was supported by the National Institute of Drug Abuse of the National Institutes of Health under T32 DA023356 and Fogarty International Center of the National Institutes of Health under Award Number D43TW009343 and the University of California Global Health Institute. Martin NK has received honoraria from Gilead, Merck, and Abbvie and was funded by the San Diego Center for AIDS Research (SD CFAR), a National Institutes of Health (NIH)-funded program (P30 AI036214) and the National Institutes of Allergy and Infectious Diseases (R01 AI147490). Skaathun B was also funded by an NIH—NIDA K01 (DA049665).

## Declaration of competing interest

The authors declare the following financial interests/personal relationships which may be considered as potential competing interests: Natasha Martin has received honoraria from Merck, Gilead, and AbbVie.

## Appendix Supplementary materials

Image, application 1
