# Supplementary material for: Differences in characteristics and interactions with close contacts among PWID in the San Diego Border Region before and during the COVID-19 pandemic
Source: J Migr Health. 2024 Sep 24;10:100267. doi: 10.1016/j.jmh.2024.100267 (PMC11471669; doi:10.1016/j.jmh.2024.100267)
Supplement: Supplementary file 1 [file mmc1.docx]

**SUPPLEMENTARY INFORMATION**

**Title**: **Differences in characteristics and interactions with close contacts among PWID in the San Diego Border Region before and during the COVID-19 pandemic**

**Authors**: Lara K Marquez, Natasha K Martin, Steffanie A Strathdee, Britt Skaathun

**Contents**

1. **Supplementary Table 1.** Number of partners pre-pandemic and during the pandemic by cross-border PWID status.
2. **Supplementary Table 2.** Proportion (%) of closest partners who inject drugs by partner type among all PWID in the San Diego Border Region.
3. **Supplementary Table 3.** Proportion (%) of closest partners who inject drugs stratified by cross-border PWID status.

**SUPPLEMENTARY TABLES**

**Table 1 Number of drug/alcohol-related, sex, and close partners pre-pandemic and during the pandemic by cross-border PWID status.** Number of partners were reported during a two-week period prior to the pandemic and the most recent two-week period during the pandemic. Cross-border PWID refers to PWID who cross the US-Mexico border to inject drugs; Non-cross-border drug user refers to people who inject drugs in their city of residence (either in San Diego [SD-PWID] or in Tijuana [TJ-PWID]) and do not cross the border to inject drugs.

| **Partner type** | **Cross-border PWID** | **San Diego PWID** | **Tijuana PWID** | **P-value** |
| --- | --- | --- | --- | --- |
|  | **Mean (SD)** | **Mean (SD)** | **Mean (SD)** |  |
| Number of drug/alcohol had contact with pre-pandemic | 3.2 (4.7) | 7.1 (15.3) | 2.4 (2.3) | 0.12 |
| Number of drug/alcohol during pandemic | 0.8 (1.6) | 0.7 (1.3) | 0.8 (1.2) | 0.17 |
| Number of sexual partners pre-pandemic | 0.5 (0.9) | 0.6 (1.0) | 0.5 (0.5) | 0.56 |
| Number of sexual partners during pandemic | 0.4 (0.6) | 0.4 (0.7) | 0.3 (0.5) | 0.94 |
| Number of partners in close contact with pre-pandemic | 2.5 (4.8) | 6.0 (13.0) | 2.5 (3.9) | 0.002 |
| Number of people no longer see because of pandemic | 1.1 (3.2) | 1.0 (2.9) | 0.9 (2.5) | 0.91 |
| Number of important partners in contact with in last 30 days (during pandemic) | 2.6 (1.8) | 3.0 (4.0) | 2.9 (1.7) | 0.15 |

**Table 2 Proportion (%) of closest partners who inject drugs by partner type among all PWID in the San Diego Border Region.** Closest partners include the five closest partners reported in the last 30 days during the pandemic. PWID: people who inject drugs.

| **Partner type** | **%** |
| --- | --- |
| Sex | 15.2 |
| Drug/alcohol | 35.0 |
| Friend | 50.0 |
| All | 78.7 |

**Table 3 Proportion (%) of closest partners who inject drugs stratified by cross-border PWID status.** Closest partners include the five closest partners reported in the last 30 days during the pandemic.

| **Partner type** | **Cross-border PWID** | **San Diego PWID** | **Tijuana PWID** |
| --- | --- | --- | --- |
| Sex | 13.2 | 16.0 | 8.2 |
| Drug/alcohol | 25.4 | 17.9 | 37.0 |
| Friend | 41.7 | 27.4 | 39.7 |
| All | 80 | 59.4 | 87 |
